# Quantitative Shear Wave Elastography: A Phantom—Based Comparison of Two Ultrasound Systems

**DOI:** 10.3390/bioengineering13020214

**Published:** 2026-02-13

**Authors:** Wadhhah Aldehani, Sarah Louise Savaridas, Luigi Manfredi

**Affiliations:** 1 Division of Population Health and Genomics, School of Medicine, University of Dundee, Dundee DD1 9SY, UK; 2478077@dundee.ac.uk (W.A.); s.savaridas@dundee.ac.uk (S.L.S.); 2 Department of Clinical Radiology, Al-Sabah Hospital, Ministry of Health, Kuwait City 13001, Kuwait; 3 Breast Screening and Imaging Department, Ninewells Hospital, Dundee DD1 9SY, UK; 4 Division of Respiratory Medicine and Gastroenterology, School of Medicine, University of Dundee, Dundee DD1 4HN, UK

**Keywords:** shear wave elastography, ultrasound phantom, breast imaging, phantom validation, quantitative elastography, tissue mimicking material, controlled probe pressure, cross-vendor comparison, measurement reproducibility

## Abstract

To evaluate cross-platform measurement consistency and diagnostic threshold requirements in shear wave elastography (SWE), this study presents a robotically controlled, phantom-based validation framework to quantify and interpret inter-vendor variability that limits clinical standardisation. A custom-fabricated polyvinyl chloride-graphite phantom containing eight spherical inclusions (15–25 mm diameter, 25–95 mm depth, 23.53–259.58 kPa stiffness), representing breast tissue mechanical properties, was evaluated using Samsung HS50 and Aixplorer ultrasound systems. Robotic automation standardised probe positioning and contact, eliminating operator-dependent variability and enabling direct, system-level comparison. Cross-platform reproducibility, accuracy against mechanically validated ground truth, and diagnostic threshold performance were assessed across 80 measurements. Both systems demonstrated excellent intra-machine reproducibility (coefficient of variation: Samsung 0.42%, Aixplorer 0.46%) with strong inter-machine correlation (r = 0.9951, *p* < 0.0001). However, systematic bias of 7.05 kPa and 95% limits of agreement spanning 38.7 kPa revealed substantial cross-platform measurement differences. All phantom inclusions (8/8) measured below their assigned ground truth stiffness on both systems, with systematic underestimation ranging from 0.33 kPa to 109.57 kPa, indicating parameter-dependent measurement distortion linked to inclusion size, depth, and stiffness. Dynamic range compression was observed (Samsung: 68.7%, Aixplorer: 59.1% of true phantom range), providing a mechanistic explanation for diagnostic threshold instability. This study contributes an interpretable validation methodology that links SWE measurement bias to physical lesion properties and imaging system characteristics, rather than relying on correlation alone. Despite strong reproducibility, the observed system-dependent bias demonstrates that SWE measurements are not directly transferable across ultrasound platforms, and system-specific collaboration is required to ensure reliable clinical interpretation.

## 1. Introduction

Shear wave elastography (SWE) is increasingly used as an adjunct to greyscale ultrasound in breast imaging, offering quantitative assessment of tissue stiffness to support clinical decision-making. It has been shown to be particularly useful in the non-invasive evaluation of presumed fibroadenomas in women aged 25 to 40 years. In these cases, low SWE stiffness values combined with characteristic ultrasound features, oval shape, circumscribed margins, and parallel orientation can, under specific criteria, safely justify the avoidance of biopsy [1]. However, the absence of standardised stiffness thresholds and documented variability in quantitative SWE outputs between ultrasound systems limits clinical adoption [2,3].

SWE measurements are affected by multiple factors, including equipment specifications, acquisition settings, probe pressure, and lesion characteristics such as size, depth, and stiffness [4,5]. The variability of SWE values across ultrasound systems undermines consistency and limits the adoption of universal stiffness thresholds [6]. This inter-machine variability also complicates both clinical interpretation and research comparisons, especially in multicentre or longitudinal patient monitoring studies. Current guidelines do not yet offer clear calibration standards or machine-specific thresholds, creating a diagnostic grey zone that may lead to patient misclassification [7].

Several phantom-based studies have evaluated the accuracy and reproducibility of SWE, but most have methodological limitations that restrict their clinical relevance [8]. Tanter et al. [9] and Mun et al. [7] assessed stiffness using manual scanning and phantoms with limited or no ground truth validation, relying instead on estimated or histopathological values. Barr et al. [10] attempted a multi-depth approach, but only a qualitative machine comparison was performed, and pressure control remained unstandardised. Hendriks et al. [11] employed an automated setup but focused solely on automated breast volume scanning (ABVS) validation, not lesion-level SWE accuracy. Moon et al. [12] and Kim et al. [2] varied inclusion geometry but lacked mechanical validation of stiffness, while Al- Mutairi et al. [13] used cryogel-based inclusions without reporting machine-to-machine variability. Across these studies, SWE was typically tested under manual conditions, with single-variable manipulations (e.g., stiffness only), and without reliable ground truth confirmation.

This study addresses the limitations of previous phantom work by introducing a custom-fabricated breast phantom containing synthetic lesions designed to replicate fibroadenomas and malignant breast masses. These lesions vary in size, depth, and stiffness. The phantom was scanned under robotic control using two ultrasound systems, Machine A (Samsung HS50) and Machine B (Aixplorer, Supersonic Imagine). By eliminating operator variability and precisely controlling geometric and physical parameters, the study isolates the influence of lesion characteristics on SWE measurements and enables direct comparison between systems under standardised conditions. The primary aim is to assess intra- and inter-machine reproducibility and determine the extent to which vendor-specific calibration is required for reliable cross-platform implementation. Measurements were systematically compared with mechanically validated ground truth stiffness values. By combining robotically standardised datasets with systematically designed targets and objective validation, this study provides the most comprehensive evaluation to date of cross-platform SWE performance, addressing both technical reproducibility and clinical implementation challenges in breast imaging.

## 2. Materials and Methods

### 2.1. Phantom Fabrication Processes

**Figure 1:**
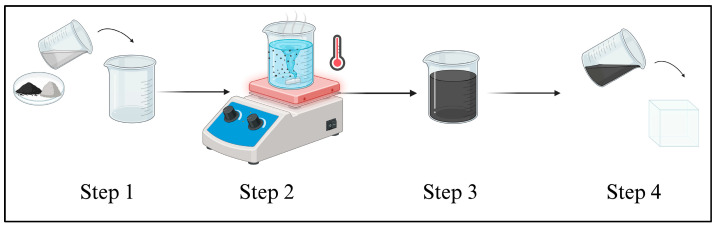
Schematic illustration of the phantom fabrication process steps. Step (1) mixing PVC, adipate and graphite powder together, Step (2) using magnetic stirrer and hotplate (Stuart, Stone, Staffordshire, United Kingdom)with 280° temperature for 30 min until polymerization occurs, Step (3) once the mixture is ready move its vacuum chamber to remove air bubbles and Step (4) Pouring the final mixture into the mould and allowing it to set at room temperature.

A PVC-based breast phantom was fabricated with embedded spherical lesions designed to evaluate the effects of size, depth, and stiffness [14]. The phantom consisted of a homogeneous polyvinyl chloride (PVC) with Di-(2-ethylhexyl) adipate and mixed with graphite powder to enhance echogenicity. As shown in Figure 1, PVC phantoms were fabricated using graphite powder as a scattering agent to enhance echogenicity.

- **Step one:** The PVC and softener were thoroughly mixed in a glass beaker and heated on a hotplate to approximately 175 °C, with frequent stirring to ensure uniform heating and homogeneous mixing.
- **Step two:** Once the mixture thickened and transitioned from a milky white to a translucent appearance, graphite powder was gradually introduced while stirring continuously to minimise clumping and ensure even dispersion throughout the matrix.
- **Step three:** Upon achieving the desired consistency, the material was removed from the heat source and placed in a vacuum chamber for a minimum of 10 min to eliminate entrapped air bubbles.
- **Step four:** The degassed mixture was then poured into the designated phantom mould. Any required internal structures or inclusions were positioned within the matrix prior to solidification. The filled mould was allowed to cool at room temperature until the material was fully set.

**Figure 2:**
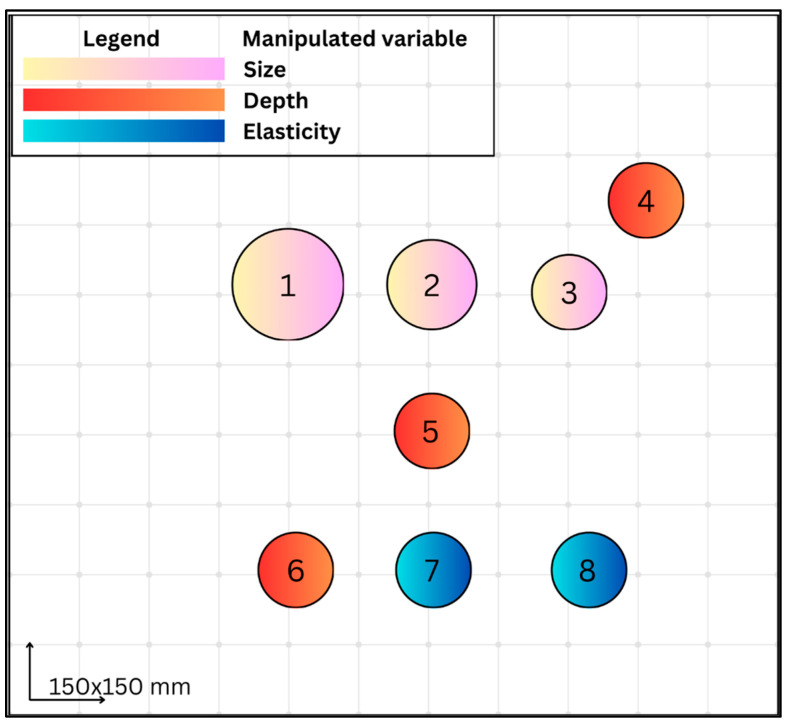
Schematic representation of the phantom design, illustrating the arrangement of eight spherical inclusions (labelled 1–8) within a 150 × 150 mm block as viewed from the anterior face.

As shown in Figure 2, eight spherical inclusions were embedded in a 150 × 150 mm phantom. Size set (Label: 1–3): three inclusions at a fixed centre depth of 45 mm, equal stiffness (52.29 kPa), with diameters 25 mm, 20 mm, and 15 mm. Depth set (Label: 4–6): three inclusions of identical geometry (15 mm diameter) and stiffness (52.29 kPa) placed at centre depths 25 mm (#4), 75 mm (#5), and 95 mm (#6). Elasticity set (Label: 6–8): three inclusions with identical geometry (15 mm diameter, 95 mm centre depth) fabricated at 52.29, 23.53, and 259.58 kPa; note that #6 is shared between the depth and elasticity sets. The intention was to fabricate inclusions with target stiffness values to mimic typical glandular breast tissue, fibroadenomas, and malignant lesions based on ranges reported in the literature [15,16,17,18]. In our breast imaging unit, a shear wave elastography stiffness threshold of 50 kPa is routinely used as part of clinical decision support when SWE is applied as an adjunct to greyscale ultrasound. The phantom inclusion stiffness values were therefore selected to span stiffness ranges below (≈23 kPa), close to (≈52 kPa), and well above (≈260 kPa) this threshold, to enable evaluation of system behaviour around a clinically relevant decision boundary, Table 1. It is acknowledged that the depth inclusions 6–8 were beyond that seen in normal clinical practice; however, this was necessary to ensure sufficient spacing to avoid acoustic overlap. The material formulations used for each inclusion and the background matrix are detailed in Table 2. Lesions were moulded separately using stiffness-controlled formulations, then embedded into the base phantom matrix prior to final curing. All ground truth stiffness values were derived from mechanical testing.

### 2.2. Mechanical Property Measurement

The elastic modulus of each formulation was measured using uniaxial compression testing. Square samples (40 × 40 × 40 mm) were fabricated from the same batches used in the phantom. Testing was conducted using an Instron 5944 universal testing machine (Shimadzu UK Ltd., Mill Court, United Kingdom) with a 500 N load cell at a constant displacement rate of 1 mm/min. The compressive stress–strain curve was generated, and Young’s modulus (E) was calculated from the linear region of the curve (5–15% strain). The mean value from three repeated tests per material was used as the ground truth stiffness.

### 2.3. Shear Wave Elastography

SWE scans were performed on two ultrasound systems: Machine A (Samsung HS50, Seoul, Republic of Korea) and Machine B (Aixplorer, Supersonic Imagine, Provence-Alpes-Côte d’Azur, France). The Samsung HS50 is a widely used mid-tier clinical platform with advanced elastography capabilities, while the Aixplorer is a reference-standard SWE system utilising UltraFast SWE technology, which represents different approaches to SWE implementation and clinical adoption patterns [19,20]. In both systems, shear wave elastography estimates tissue stiffness by measuring the propagation speed of mechanically induced shear waves within the medium. The shear wave speed is converted by the system software into an elastic modulus (reported in kPa) using the relationship $E\;=\;3pc_{s}^{2}$, assuming linear elastic, isotropic material behaviour, where p is material density and${\;c}_{s}$, is shear wave speed. All reported SWE values correspond to system-derived modulus estimates within the defined ROI.

**Figure 3:**
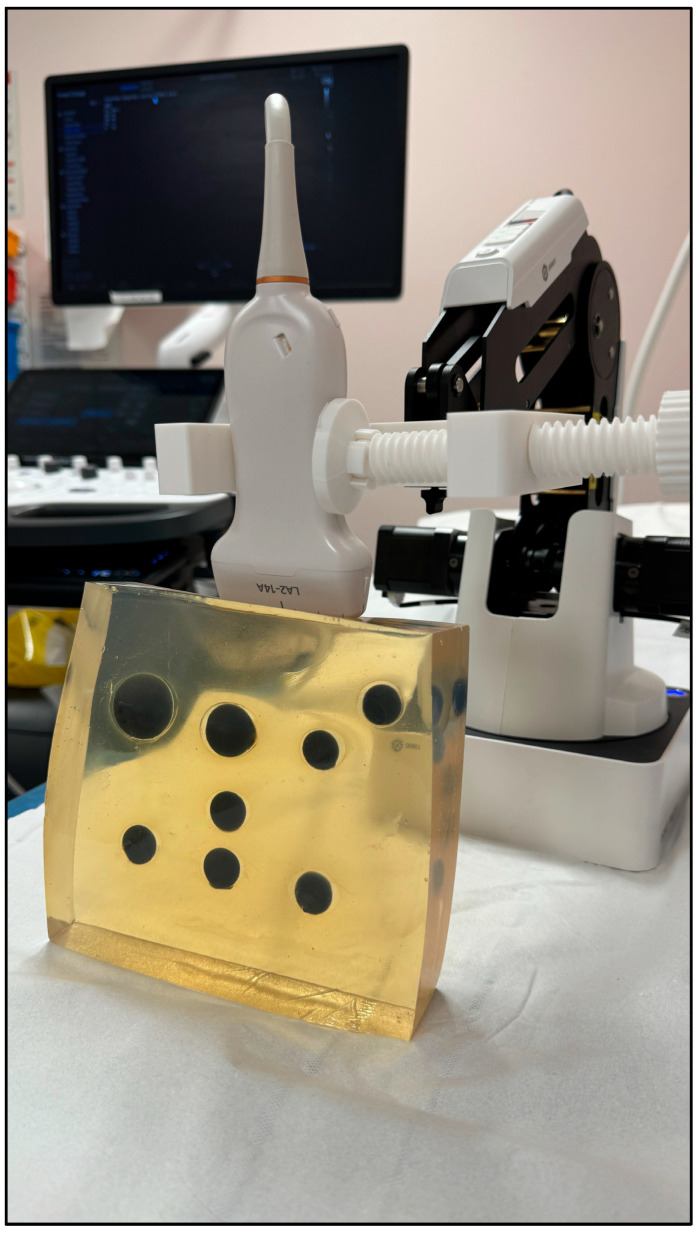
During shear wave elastography scanning on machine A, a robotic arm is used to ensure consistent positioning, orientation, and contact pressure of the ultrasound probe. The robotic arm controls both vertical and lateral movements of the probe, eliminating operator variability.

Identical scanning presets were applied to both systems: SWE mode with depth range: 0–120 mm using a 4–15 MHz probe. For each inclusion, the focal zone and display depth were adjusted according to the inclusion depth range (25–95 mm) to maintain adequate acoustic penetration throughout the phantom. Figure 3 is the scanning setup.

**Robotic Automation system:** A robotic arm, Dobot Magician, was used to position and maintain the transducer throughout the scanning process, ensuring consistent probe positioning and orientation across all measurements. The primary objective of robotic automation was to eliminate operator-dependent variability in probe handling, positioning, and scanning path.

This collaborative robotic arm offers five degrees of freedom (DOFs), enabling translational movement along the *X*, *Y*, and *Z* axes, as well as controlled rotation around the *X*-axis (pitch) and *Y*-axis (yaw) of the end-effector. Control is achieved via a USB using Dobot’s proprietary API (Application Programming Interface), which enables scripted Cartesian path, with sub-millimetre positional repeatability (<0.2 mm).

**The scanning protocol:** The shear wave elastography methodology published by Evans et al. [1], for breast lesion assessment was followed. For each scan, both B-mode and SWE images were obtained in two orthogonal planes. Images were allowed to stabilise for approximately 10 s before measurements were taken. A 2 mm circular region of interest (ROI) was then manually positioned over the stiffest region of the elastogram, with the mean SWE value within the ROI recorded in kilopascals (kPa) [21]. The average SWE measure was then calculated. Each lesion was scanned five times on each ultrasound system over a two-week period.

### 2.4. Statistical Analysis

All analyses were conducted using RStudio (v4.2.2). Intra-machine reproducibility was assessed using the coefficient of variation (CV) calculated for each inclusion within each system. Inter-machine correlation was evaluated using Pearson correlation analysis. Cross-platform agreement was assessed using Bland–Altman analysis with calculation of mean bias and 95% limits of agreement. Mean absolute error (MAE) and root mean square error (RMSE) were computed as additional agreement metrics.

Parameter dependencies were analysed using Kruskal–Wallis testing for size effects across inclusion diameters. Depth dependency was assessed through Pearson correlation analysis between measurement depth and measurement bias. Dynamic range analysis compared measured versus true stiffness ranges across systems.

## 3. Results

Both ultrasound systems demonstrated exceptional intra-machine reproducibility under controlled robotic scanning conditions. Intraclass correlation coefficients approached perfect reliability (ICC = 0.999 for Machine A, ICC = 0.998 for Machine B), reflecting the elimination of operator variability through robotic positioning and the consistent mechanical properties of the PVC phantom materials. Inter-machine reproducibility was also excellent (ICC = 0.968, 95% CI: 0.89–0.99), indicating strong agreement in relative stiffness rankings between systems.

However, substantial differences were observed in absolute SWE measurements compared to mechanical ground truth values Table 3. Under the controlled laboratory conditions, Machine A demonstrated superior accuracy with a mean absolute error (MAE) of 13.74 ± 26.52 kPa compared to Machine B’s MAE of 20.79 ± 36.18 kPa.

**Figure 4:**
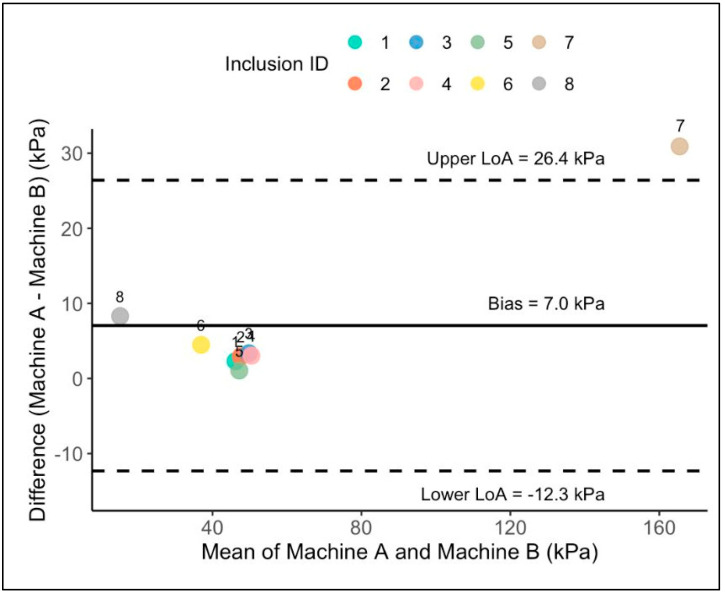
Bland–Altman plot comparing shear-wave elastography measurements between Machine A (Samsung HS50) and Machine B (Aixplorer). The *x*-axis shows the mean stiffness of the two systems, and the *y*-axis shows their difference (Machine A–Machine B). Solid line represents mean bias (7.05 kPa); dashed lines indicate 95% limits of agreement (–12.30 to 26.40 kPa). Data points are colour-coded by inclusion ID (1–8).

Bland–Altman analysis demonstrated a systematic inter-machine bias of 7.05 kPa, with Machine A consistently measuring higher than Machine B across all inclusion types, Figure 4. The 95% limits of agreement ranged from −12.30 to +26.40 kPa, indicating that the difference between machines could vary by up to 38.7 kPa for individual measurements. From a clinical perspective, a cross-platform difference of up to 38.7 kPa is substantial relative to commonly used shear wave elastography decision thresholds for breast lesion characterisation, which typically lie in the range of 40–50 kPa. At this scale, a lesion measured near a diagnostic cut-off on one system could be classified differently on another system despite identical physical properties. This finding highlights the potential risk of systematic misclassification when absolute SWE thresholds are applied interchangeably across vendors without calibration.

The strongest agreement between machines was observed for the shallow fibroadenoma inclusion (Inclusion 4 at 25 mm depth). In contrast, the largest inter-machine discrepancy was observed for the malignant inclusion at maximum depth (Inclusion 7 at 95 mm depth, difference = 30.91 kPa), a depth that exceeds those typically encountered in routine clinical practice. This trend has clinical significance, showing that agreement between machines is highest at typical breast imaging depths (≤40 mm), where measurement reliability is crucial for diagnosis. At these clinically relevant depths, the mean absolute difference was 3.04 kPa, compared to 7.62 kPa at greater depths, indicating that cross-platform variability mainly impacts extreme cases that are uncommon in everyday practice.

Inclusion size effects were evaluated using three inclusions at the same depth (45 mm) and stiffness (52.29 kPa) with varying diameters. Inclusion size demonstrated a strong negative correlation with measurement accuracy for both systems (Machine A: r = −0.997, *p* = 0.051; Machine B: r = −0.989, *p* = 0.095). The 15 mm diameter inclusion (#3) achieved the highest accuracy with machine A, MAE = 0.85 kPa, and machine B, MAE = 4.21 kPa. The 25 mm diameter inclusion (#1) showed the largest deviation with machine A MAE = 4.99 kPa, while machine B MAE = 7.29 kPa. The 20 mm diameter inclusion (#2) demonstrated intermediate accuracy with machine A MAE = 3.21 kPa and machine B MAE = 6.15 kPa, Table 3.

Temporal stability analysis over the 2-week measurement period revealed excellent consistency, with coefficient of variation (CV) values below 1% for 15 of 16 machine-inclusion combinations. Only one inclusion (Machine B, Inclusion 8) showed significant temporal drift (*p* = 0.047), likely reflecting measurement challenges at very low stiffness levels (23.53 kPa) combined with significant depth (95 mm).

Depth effects were more pronounced, with both machines showing significant measurement degradation at greater depths. Within clinically relevant depths ≤ 40 mm, both systems maintained acceptable accuracy: machine A showed minimal underestimation of 0.6% (MAE 0.33 kPa) at 25 mm depth, while machine B demonstrated 6.4% underestimation (MAE 3.37 kPa). However, at 95 mm depth, Machine A underestimated stiffness by 25.1% while Machine B showed 33.6% underestimation compared to ground truth values. The malignant inclusion at this extreme depth (Inclusion 7) exhibited errors of 78.66 kPa and 109.57 kPa, respectively, Table 3.

These findings show that both systems are highly reproducible under controlled conditions. However, their absolute stiffness measurements differ systematically, meaning the systems are not directly interchangeable, requiring machine-specific calibration or correction factors. The robotic scanning protocol eliminated operator-dependent variability, enabling precise quantification of inherent system differences that would otherwise be obscured by manual scanning inconsistencies.

## 4. Discussion

What distinguishes this study is the integration of a robotic arm to standardise transducer position and a phantom design with precisely varied inclusion parameters. This setup enables rigorous, reproducible assessment of how specific lesion characteristics alter SWE readings, eliminating operator variability, a limitation identified in previous investigations [22,23]. The robotic system demonstrated excellent measurement repeatability (average CV = 0.44%, maximum CV = 1.62%), representing substantial improvement over conventional validation approaches, where operator-dependent factors can introduce significantly higher measurement variability [24]. This automated approach eliminated operator-dependent positioning errors while providing consistent probe placement.

However, while the robotic system maintained consistent probe positioning, direct contact pressure was not quantitatively measured. Yet, because the probe was positioned at the same predefined height and position on the phantom for all acquisitions, the applied contact conditions and probe pressure were consistent and comparable across measurements. This approach achieved excellent reproducibility and enabled systematic comparison between systems, although the contact conditions may differ from typical clinical scanning scenarios. This methodological consideration should be evaluated in the context of the study’s primary objective: systematic characterisation of inter-system measurement differences under standardised conditions.

The stability of the phantom properties over the two-week measurement period is primarily attributable to the material composition. The PVC-based formulations used are non-aqueous and chemically stable, which limits dehydration, diffusion, and deformation effects that commonly affect hydrogel-based phantoms. In addition, the use of plasticiser-modified PVC results in stable mechanical behaviour under standard laboratory conditions. As a result, no measurable change in stiffness was observed over the two-week storage and measurement period, allowing observed variations to be attributed to system-specific measurement behaviour rather than phantom degradation [25,26].

This study confirms that while both systems generate highly reproducible SWE measurements under controlled conditions (CV ≤ 1.6%), there are substantial differences in their agreement with ground truth values. Both systems demonstrated excellent correlation with reference standards (r > 0.98) but exhibited distinct bias patterns. Machine A showed superior accuracy compared to Machine B under our experimental conditions. These phantom-specific findings highlight the importance of system characterisation but should not be extrapolated to clinical performance comparisons. Clinical accuracy depends on multiple factors, including tissue heterogeneity, operator technique, and patient-specific variables, which cannot be captured in controlled phantom studies.

Our findings align with previously published work demonstrating vendor-specific variability in SWE outputs. Javed et al. observed significant inter-system reliability differences, with some system pairs showing ‘almost perfect’ agreement while others demonstrated ‘poor to moderate’ reliability when compared across vendors. Systematic reviews have consistently identified inter-vendor differences as a barrier to clinical standardisation, with ongoing efforts by organisations such as QIBA working to reduce measurement variability between systems [23,27,28]. Our controlled phantom validation provides quantitative evidence that these differences persist even under optimal measurement conditions.

From a clinical perspective, dynamic range compression may reduce the apparent separation between benign and malignant lesions by systematically underestimating higher stiffness values. For example, a stiff malignant lesion that exceeds typical diagnostic thresholds may be measured closer to intermediate stiffness ranges, potentially overlapping with values observed in benign entities such as fibroadenomas. In practice, this compression could reduce confidence in threshold-based decision-making, particularly for lesions near biopsy cut-offs, and may contribute to false-negative assessments or increased reliance on adjunct imaging features. Our findings highlight that strong correlation alone does not guarantee preserved diagnostic contrast across the full stiffness spectrum.

Beyond the Bland–Altman analysis, the observed bias patterns were systematic and consistent across all stiffness ranges. This suggests that regression-based calibration or correction models could be used to improve cross-platform comparability. Calibration curves derived from mechanically validated reference phantoms could map measured SWE values to system-specific corrected stiffness estimates. Although such correction methods were beyond the scope of this study, the quantified bias and dynamic range compression provide a basis for future development and validation of regression-based calibration strategies.

In current breast imaging practice, shear wave elastography is used to provide supportive quantitative information alongside grayscale ultrasound, with measurements interpreted within the same ultrasound system. Under controlled conditions, both systems demonstrated excellent intra-system reproducibility, supporting the use of SWE as a source of consistent quantitative information alongside greyscale ultrasound. However, systematic inter-system bias and wide limits of agreement indicate that absolute SWE stiffness values are not interchangeable across platforms, limiting the application of uniform SWE cut-off values and supporting the need for system-specific interpretation or calibration [27,28].

Consistent with known principles of shear wave attenuation [26], the analysis revealed strong correlations between measurement depth and bias magnitude (r = 0.9455–0.9890) or depth-dependent measurement degradation. However, the clinical relevance of this finding requires careful interpretation within breast imaging practice. Our study examined depths from 25 to 95 mm, with the most noticeable effects at 95 mm, a level that significantly exceeds typical clinical scenarios. In routine breast imaging, most lesions occur within 20–40 mm of the skin surface [29,30], corresponding to the depth range where both systems demonstrated superior accuracy. Future validation studies should prioritise depth ranges representative of clinical practice to establish clinically relevant performance benchmarks.

Although inclusion size demonstrated a statistically significant association with SWE measurements (*p* < 0.002), the clinical significance requires careful evaluation. Our analysis revealed systematic bias patterns ranging from 1.6% to −13.9% across the size range studied, with smaller inclusions consistently demonstrating better accuracy. The statistical significance likely reflects the precision of our controlled experimental conditions rather than clinically meaningful differences, supporting previous investigations showing SWE measurements remain clinically consistent across various lesion sizes when standardised protocols are employed [12,31].

This framework provides a reproducible approach for multicentre SWE standardisation that can support consistent measurement across clinical sites. The robotic approach enables standardised quality assurance protocols, while the systematic phantom design provides reference standards for cross-platform calibration. In future work, the robotic measurement protocol could support multi-centre quality assurance using a shared reference phantom and predefined scanning scripts. Identical acquisition protocols could be applied across institutions to compare SWE performance under controlled conditions, independent of operator variability. This would enable assessment of system stability, inter-machine bias, and temporal drift. Such an approach supports long-term QA monitoring and cross-site benchmarking and provides a practical framework for standardising SWE measurements across clinical networks.

## 5. Limitations

There are several limitations that should be acknowledged. First, although the robotic system maintained consistent probe positioning, direct contact pressure could not be quantitatively measured due to the absence of an integrated pressure sensor. However, the probe was deployed to the same predefined height and position on the phantom for all acquisitions. This resulted in consistent and comparable contact conditions across measurements, although these conditions may not fully reflect typical clinical scanning scenarios. This limitation should be considered in the context of the study’s primary objective, which was to characterise inter-system measurement differences under standardised conditions. Future studies could incorporate an inline force or load sensor within the robotic end-effector to directly quantify and control transducer contact pressure, enabling closer simulation of clinical scanning conditions.

Second, while robotic positioning eliminated variability in probe handling, region-of-interest (ROI) placement was performed manually by targeting the stiffest region within the elastogram, in accordance with established clinical practice. This approach introduces potential selection bias, particularly in heterogeneous regions. Automated or semi-automated ROI placement strategies, such as algorithm-based stiffness maximisation or fixed-grid sampling, could reduce subjectivity and improve measurement consistency and should be explored in future studies.

Third, the inclusion of extreme target depths (up to 95 mm) exceeds typical clinical scenarios in breast imaging, where most lesions are encountered within 20–40 mm of the skin surface. As a result, findings at greater depths have limited direct clinical applicability and were included primarily to characterise system behaviour under challenging conditions.

A further limitation is that only two ultrasound systems were evaluated. While this restricts direct generalisation across vendors, the use of a controlled, standardised comparative study design enables robust characterisation of inter-system behaviour under identical conditions [11,25]. This approach reflects established methodologies used in phantom-based ultrasound elastography validation to isolate system-specific performance characteristics. Extension of this design to additional vendors and SWE implementations would be required to support broader standardisation.

Finally, although the phantom materials were mechanically validated to match target stiffness values, they do not fully replicate the complex viscoelastic behaviour of in vivo breast tissue. Biological tissues exhibit heterogeneity, anisotropy, and time-dependent responses, including frequency-dependent shear wave propagation and nonlinear mechanical behaviour, which are not fully captured by homogeneous phantom materials. Consequently, absolute stiffness values obtained in phantom studies should be interpreted as system-specific performance benchmarks rather than direct surrogates for in vivo tissue behaviour. This limitation is inherent to phantom-based validation studies and highlights the importance of complementary clinical validation.

## 6. Conclusions

This study demonstrates that shear wave elastography provides excellent intra-system reproducibility under controlled conditions; however, cross-platform agreement is limited by operator-dependent factors, parameter-specific measurement behaviour, absence of validated reference standards, and system-specific threshold effects.

Our findings should not be applied to clinical diagnostic decisions. Rather, they define the technical limitations that constrain cross-vendor comparability and highlight the need for calibration and standardisation. The quantified bias patterns and dynamic range effects identified in this work provide a basis for system-specific correction, quality assurance, and performance benchmarking.

By integrating robotic acquisition, mechanically validated phantom reference standards, and systematic cross-platform analysis, this study establishes a reproducible framework for SWE system evaluation. This approach addresses key reproducibility challenges that currently limit the broader clinical harmonisation of quantitative elastography and supports the development of vendor-aware calibration pathways for future multi-centre implementation.

## Figures and Tables

**Table 1:** Relationship between phantom inclusion stiffness ranges and commonly used clinical SWE diagnostic categories.

Inclusion Type (Phantom)	Ground Truth Stiffness (kPa)	Typical Clinical Interpretation
**Cyst-like inclusion**	~23 kPa	Benign/low stiffness
**Fibroadenoma-like**	~52 kPa	Benign/borderline
**Malignant-like**	~260 kPa	Highly suspicious malignant

**Table 2:** Material compositions weight% for each phantom inclusion and background. Inclusion numbers correspond to the schematic in Figure 2.

Lesion Type	PVC	Softener	Graphite Powder	Inclusion
Fibroadenoma	10	88	2	(1–6)
Cyst	8	91.5	0.5	(7)
Cancer	12	86	2	(8)
Background	7.5	92.5	0	-

**Table 3:** Phantom inclusion characteristics and measurement accuracy showing mean ± standard deviation (SD) of shear wave elastography measurements and mean absolute error (MAE) for each ultrasound system compared to mechanical ground truth values.

Inclusion ID	Depth (mm)	Size (mm)	Ground Truth kPa	Machine A Mean ± SD (kPa)	Machine B Mean ± SD (kPa)	MAE A	MAE B
***1***	45	25	52.29	47.30 ± 0.30	45.00 ± 0.07	4.99	7.29
***2***	45	20	52.29	49.08 ± 0.16	46.14 ± 0.15	3.21	6.15
***3***	45	15	52.29	51.44 ± 0.15	48.08 ± 0.22	0.85	4.21
***4***	25	20	52.29	51.96 ± 0.15	48.92 ± 0.08	0.33	3.37
***5***	75	20	52.29	47.72 ± 0.11	46.66 ± 0.23	4.57	5.63
***6***	95	20	52.29	39.18 ± 0.23	34.70 ± 0.12	13.11	17.59
***7***	95	20	259.58	180.92 ± 0.13	150.01 ± 0.11	78.66	109.57
***8***	95	20	23.53	19.32 ± 0.18	11.02 ± 0.18	4.21	12.51

## Data Availability

The original contributions presented in this study are included in the article. Further inquiries can be directed to the corresponding author.
